# EGR1 Promotes Erastin-induced Ferroptosis Through Activating Nrf2-HMOX1 Signaling Pathway in Breast Cancer Cells

**DOI:** 10.7150/jca.95328

**Published:** 2024-06-24

**Authors:** Zhirong Lin, Zifei Liu, Zhilong Pan, Yunyi Zhang, Xinyu Yang, Yaxin Feng, Ruihua Zhang, Wenfeng Zeng, Chang Gong, Jianing Chen

**Affiliations:** Breast Tumor Center, Sun Yat-Sen Memorial Hospital, Sun Yat-Sen University, Guangzhou, 510120, China.

**Keywords:** EGR1, breast cancer, ferroptosis, prognosis.

## Abstract

**Purpose:** Early growth response 1 (EGR1) is a crucial transcription factor composed of zinc finger structures, inhibitory and activating regulatory regions. We identified the biological effect and molecular mechanisms of EGR1 in breast cancer (BC).

**Methods:** We used qRT-PCR, western blot and immunohistochemistry to examine the expression of EGR1 in BC samples. CCK-8 and colony assay were performed to reveal the effect of EGR1 on the proliferation of BC cells. LDH release assay, MCB assay, MDA assay, C-AM assay and TMRE assay were performed to measure the levels of LDH release, GSH, MDA, LIP and mitochondrial membrane potential. The regulation of EGR1 on the expression of Nrf2 and HMOX1 was investigated through Western blot. Xenograft models were conducted to determine the impact of EGR1 overexpression on BC *in vivo*.

**Results:** The expression of EGR1 was downregulated in BC tissues compared with the normal tissues, and lower expression of EGR1 associated with poorer clinical outcome in BC patients. Through *in vitro* experiments, we found that EGR1 downregulation facilitated the proliferation of BC cells, and overexpression of EGR1 inhibited the proliferation of BC cells. In addition, EGR1 knockdown alleviated erastin-induced ferroptosis and overexpression of EGR1 facilitated erastin-induced ferroptosis in BC cells. Moreover, overexpression of EGR1 facilitated the anti-tumor effect caused by erastin *in vivo*. Mechanistically, the phosphorylation levels of Nrf2 and the expression of HMOX1 were reduced due to the downregulation of EGR1, and increased due to the upregulation of EGR1. Additionally, the finding that EGR1 facilitated erastin-induced ferroptosis was alleviated by the inhibition of Nrf2-HMOX1.

**Conclusion:** The expression of EGR1 is downregulated in BC, which is correlated with poor prognosis of BC patients. EGR1 suppresses the proliferation of BC cells and facilitates erastin-induced ferroptosis by activating Nrf2-HMOX1 signaling pathway in BC cells.

## Introduction

Breast cancer (BC) is a common disease worldwide, posing a serious global health threat 1. According to statistics, there were approximately 2.3 million new cases of BC and about 680,000 deaths caused by BC in 2020, which indicates that BC has become one of the most common and dangerous cancers 2. Although some important progress has been made in BC research 3-5, the 5-year survival rate is only about 90%, which is still unsatisfactory 6. Therefore, to further improve the prognosis of BC patients, more effective treatments need to be found.

Ferroptosis, an iron-dependent programmed cell death mediated by lipid peroxidation, is an important tumor suppression mechanism and plays a crucial role in tumor treatment 7, 8. Several biomarkers, such as GPX4 and p53, have been identified for their regulatory effect on ferroptosis, and some compounds targeting these molecules to induce ferroptosis have already been found 9, 10. ATF3, an important transcription factor, promoted erastin-induced ferroptosis by regulating SLC7A11 11. However, tumors evolved many mechanisms to evade ferroptosis to facilitate tumor development 12. Therefore, there is an urgent need to identify more effective ferroptosis driver to inhibit the progression of tumors.

EGR1, a member of the EGR family, is an important transcription factor composed of zinc finger structures, inhibitory and activating regulatory regions, which can specifically bind to target gene sequences and regulate their transcription 13. EGR1, predominantly located in the nucleus, is involved in crucial processes including cell proliferation, growth, apoptosis and ferroptosis through various signaling pathways 14, 15. There is mounting evidence indicating the association between EGR1 and cancer. For example, EGR1 promoted the expression of Smad7 by binding to the promoter of NDRG1, thereby inhibiting the EMT process in bladder cancer 16. In addition, EGR1 inhibited the activation of AKT signaling pathway by translocation to the nucleus from cytoplasm, thereby inhibiting the advancement of liver cancer 17. Moreover, EGR1 induces ferroptosis via MAP3K14-NF-κB axis to promote intervertebral disc degeneration, implicating the roles of EGR1 in the regulation of ferroptosis 18. However, it is still unclear whether regulating EGR1 is an effective strategy for the treatment of BC. Considering this situation, our study focused on the role of EGR1 in BC.

In our study, we analyzed public databases and conducted experiments using our own clinical samples to identify the association between the expression of EGR1 and the prognosis of BC patients. Our study revealed significant reduction in the expression of EGR1 in BC compared with normal tissues. Moreover, we demonstrated its correlation with patient prognosis. Additionally, our study indicated that EGR1 inhibited the growth of BC cells and facilitated erastin-induced ferroptosis through activating Nrf2-HMOX1 signaling pathway.

## Materials and Methods

### Cell culture

Normal breast epithelial cells MCF-10A and six human BC cell lines (MCF-7, ZR-75-1, BT-474, SK-BR3, MDA-MB-231, MDA-MB-468) were purchased from the Cell Bank of the Chinese Academy of Sciences (Shanghai). All cells were cultured in suitable medium containing 10% fetal bovine serum (Gibco), 100 U/mL penicillin and 100 μg/mL streptomycin and maintained in a humidified atmosphere of 5% CO^2^ at 37°C.

### Patient specimens

BC tissues and matched normal tissues were obtained from Sun Yat-sen Memorial Hospital, Sun Yat-sen University between 2020 and 2021 (n=50). The inclusion criteria were as follows: (1) patients who had undergone breast cancer radical surgery with confirmed postoperative pathology indicating invasive ductal carcinoma; (2) absence of clear tumor invasion of surrounding organs as demonstrated by preoperative chest X-ray, ultrasound and CT scan; (3) no prior history of radiotherapy or chemotherapy before surgery. Exclusion criteria were as follows: (1) exclusion of patients with infectious diseases such as acute mastitis; (2) exclusion of patients with other malignant tumors; (3) exclusion of patients with autoimmune diseases such as rheumatoid arthritis; (4) Pregnant and lactating women were excluded. The research protocols involving the use of patient specimens were approved by the Ethics Committee of Sun Yat-sen Memorial Hospital, Sun Yat-sen University. All clinical samples used were collected with informed consent from the patients.

### RNA extraction and qRT-PCR Assay

TRIzol reagent (Invitrogen, USA) was used to extract total RNA from tissues and cultured cells. The Prime Script RT Reagent Kit (Takara, Japan) was used to synthesize complementary DNA (cDNA), and qRT-PCR was performed using the SYBR Green Premix Ex Taq (Takara, Japan) on the LightCycler480 instrument (Roche) according to standard protocols. The sequences of primers for the qRT-PCR are listed in Table S1. Based on the expression of GAPDH, the relative expression levels of EGR1 were calculated using the 2^-ΔΔCt^ method.

### Protein extraction and Western blot Assay

The total protein extraction of tissues and cultured cells was performed using RIPA buffer (Beyotime, China) containing phosphatase inhibitor and protease inhibitor (Beyotime, China). The lysates were centrifuged at 1.2 × 10^4^ g, 4°C for 20 minutes. Protein concentrations were determined using the BCA protein assay kit (Thermo, USA). Equal amounts of proteins were separated by SDS-PAGE and transferred onto PVDF membranes (Millipore, USA). The membranes were incubated with primary antibodies overnight at 4°C. After that, the membranes were washed with TBST and incubated with secondary antibodies (Cell Signaling Technology, USA). The primary antibodies used were as follows: EGR1 (abcam, ab194357), p-Nrf2 (abcam, ab76026), Nrf2 (abcam, ab62352), HMOX1 (abcam, ab68477) and β-actin (proteintech, 81115-1-RR).

### Immunohistochemistry

After the patients' tumor tissues were embedded in paraffin, sectioned into 4μm-thick sections, deparaffinized and antigen-retrieved, they were incubated overnight at 4°C with specific primary antibodies against EGR1 or Ki67. Subsequently, the sections were incubated with secondary antibodies at room temperature for 1 hour, followed by measurement using DAB. The staining intensities were graded as follows: 0, negative; 1, weak positive; 2, moderate positive; 3, strong positive. The IHC scores were determined by multiplying the staining intensity scores with the percentage of positive cells. The primary antibodies used were as follows: EGR1(abcam, ab194357) and Ki67(ZM-0167).

### Plasmid constructions and cell transfection assay

BC cells with 80% confluence in 6-well plates were transfected with control siRNA or EGR1 siRNA using Lipofectamine 2000 (Invitrogen, USA) according to the manufacturer's protocol. The negative control used non-specific oligonucleotides that did not complement any human genes. The sequences of the two targeting siRNAs for EGR1 are shown in Table S2. All siRNAs were synthesized by Aiji Biotech Company in Guangzhou, China. To overexpress EGR1, the EGR1 cDNA was cloned into the vector pLVX-FLAG-puro. For lentivirus infections, the cells were cultured in 24-well plates for infection by virus containing medium with polybrene, incubated at 37 °C for 24 h and then replaced by suitable medium with 10% FBS.

### CCK-8 and colony formation assays

CCK-8 assay involved the enzymatic digestion of 1000 cells for each well and seeding them in a 96-well plate. 10 μl of CCK-8 solution (Apexbio, USA) was added to each well and the plate was incubated at 37°C for 2h before measuring the absorbance at 450 nm once a day for a total of 4 days. For colony formation experiments, 1000 cells for each well were seeded in a 6-well plate and cultured for 10 days. Then, the cells were fixed with 4% paraformaldehyde and stained with 0.1% crystal violet. The number and size of colonies containing more than 50 cells were detected. All experiments were performed in triplicate.

### Lactate dehydrogenase release assay

The lactate dehydrogenase (LDH) release assay was performed according to the manufacturer's protocol (Beyotime, China). The amount of color formed is proportional to the number of lysed cells. Cells were seeded in a 96-well plate. Then erastin (1μM) were added separately. In some assay, ZnPP (5μM) was added. After 24h, LDH levels were determined by analyzing the amount of LDH released into the cell culture supernatant. Absorbance signals at 490 nm were obtained using a microplate reader. To determine the percentage of LDH release, the experimental LDH release quantities were calculated relative to the control LDH release quantities, as stated in the provided instructions.

### Measurement of glutathione

Glutathione (GSH) was determined using monochlorobimane (MCB) (Sigma-Aldrich, USA). Cells were plated in a black 96-well plate. After the treatment mentioned above, cells were incubated at 37°C for 30 minutes with 32µM MCB (PBS). Fluorescence measurement was performed using Victor Nivo 5S with excitation set at 390 nm and emission set at 478 nm.

### Measurement of MDA

The measurement of cellular malondialdehyde (MDA) was conducted using the MDA assay kit (Beyotime, China) to determine the MDA content in cells. In simple terms, cells were seeded into a 6-well plate and cultured overnight. After the treatment mentioned above, the cells were collected and lysed. After protein quantification, the lysates were added with MDA working solution and heated at 95°C for 15 minutes. After centrifuging at 1000 rpm for 10 minutes at 4°C, the supernatant was measured at 532 nm using Victor Nivo 5S. The concentration of MDA is calculated using a standard curve.

### Determination of the labile iron pool

Calcein-acetoxymethyI ester (C-AM) assay was adopted to detect cellular labile iron pool. After the treatment mentioned above, the cells were washed twice with PBS and then incubated with 2µM calcein-acetoxymethyI ester (GLPBIO, USA) at 37°C for 30 minutes. Subsequently, the cells were washed with PBS and incubated with or without 5µM deferoxamine at 37°C for 1 hour. Flow cytometry was used to analyze the cells. The levels of the labile iron poor were determined by the difference between cellular mean fluorescence with and without deferoxamine incubation.

### Mitochondrial membrane potential assay

Mitochondrial membrane potential was determined using TMRE (MCE, China) staining. In short, when mitochondrial membrane potential is high, TMRE accumulates in the mitochondria and produces red-orange fluorescence. Cells were seeded in a 96-well plate. After the treatment mentioned above, the cells were incubated with 1µM TMRE in PBS at 37°C for 30 minutes. The fluorescence intensity was measured using Victor Nivo 5S with an excitation wavelength of 540 nm and an emission wavelength of 595 nm.

### *In vivo* xenograft models

Female nude mice (4-5 weeks old) were purchased from Experimental Animal Center of Sun Yat-sen University (Guangdong, China). All animal experiments were approved by the Institutional Animal Care and Use Committee of the Sun Yat-Sen University. For the xenograft models, 1 × 10^6^ MDA-MB-231 cells with or without EGR1 overexpression were injected into each mouse (5 mice per group). Mice were treated with or without erastin (i.p., 20 mg/kg, once every other day) and ZnPP (i.p., 10 mg/kg, once every other day). Tumor volume (V = length × width^2^ × 0.5) was monitored every 3 days and tumor weight was measured at the end of the experiment.

### Statistical analysis

Statistical analysis was conducted using GraphPad Prism 8.0 software. All cell experiments were repeated at least three times. Student's t-test was used for comparisons between two groups. Multi-group's comparisons were performed by one-way ANOVA. The correlations between IHC scores and clinicopathologic characteristics were using chi-square test. Survival analysis was performed using the Kaplan-Meier method. P value < 0.05 was considered statistically significant for differences. The significance levels were denoted as follows: *p < 0.05, **p < 0.01, ***p < 0.001, ****p < 0.0001.

## Results

### EGR1 was downregulated in BC tissues and correlated with prognosis in BC patients

**Figure 1:**
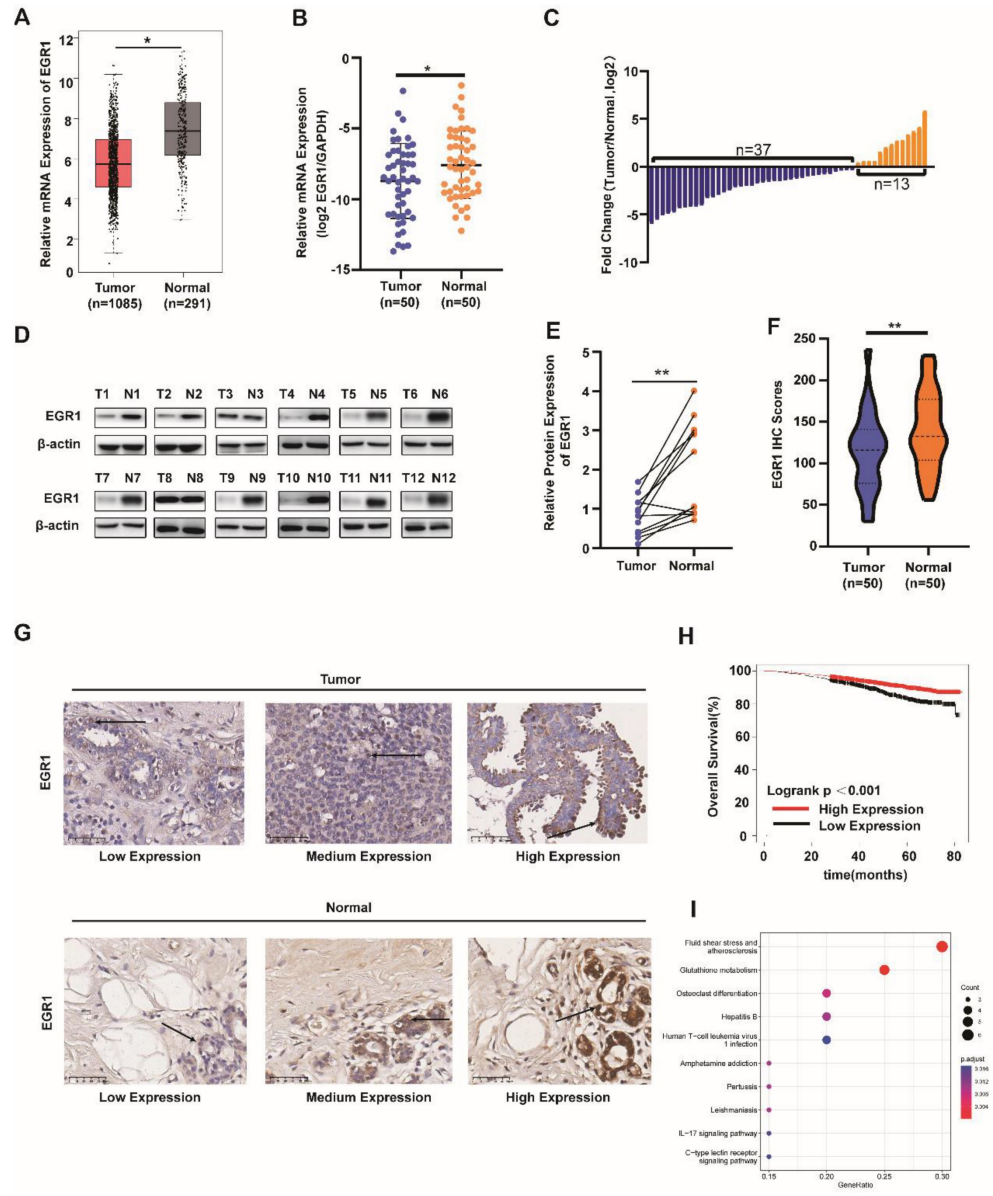
** EGR1 was downregulated in BC tissues and correlated with prognosis in BC patients. (A)** mRNA levels of EGR1 in BC and normal breast tissues in the TCGA database. **(B, C)** mRNA levels of EGR1 in cancer tissues and adjacent tissues of BC patients (n=50) in Sun Yat-sen Memorial Hospital. **(D, E)** Western blot analysis of protein expression levels of EGR1 in cancer tissues and adjacent tissues of BC patients in Sun Yat-sen Memorial Hospital. **(F, G)** Statistical analysis and representative images of IHC staining of EGR1 in BC and normal breast tissues from Sun Yat-sen Memorial Hospital. The arrow indicates that EGR1 is predominantly located in the cell nucleus. **(H)** The association between EGR1 expression and overall survival in BC patients using K-M plotting based on the TCGA database. **(I)** KEGG pathway analysis of the genes significantly associated with the EGR1 expression in BC from R2 database.

Firstly, we analyzed the mRNA levels of EGR1 in BC tissues and normal tissues using the TCGA database. We found that EGR1 was significantly downregulated in tumor tissues compared with normal tissues (Figure 1A). Then, we validated this result using our own samples, demonstrating that the mRNA levels of EGR1 were decreased in 37 out of 50 BC patients (Figure 1B and C). Subsequently, by western blot and immunohistochemistry, we confirmed the lower expression of EGR1 protein in tumor tissues compared with normal tissues (Figure 1D-G).

As shown in Table 1 about the detailed clinic parameters of enrolled patients, we found that the expression of EGR1 was significantly correlated with TNM stage, subtype and Ki-67 level. Then we employed the Kaplan-Meier survival analysis (K-M curve) to examine the association between EGR1 expression and overall survival. We found that BC patients with higher EGR1 expression had longer overall survival (Figure 1H). To identify the effects of EGR1 on BC, we analyzed data from R2 database and carried out GO and KEGG analysis, revealing that EGR1 was involved in the glutathione metabolism pathway which was associated with proliferation and ferroptosis (Figure 1I). Collectively, low expression of EGR1 in BC tissues may be associated with an increased risk.

### Knockdown of EGR1 facilitated the proliferation of BC cells

**Figure 2:**
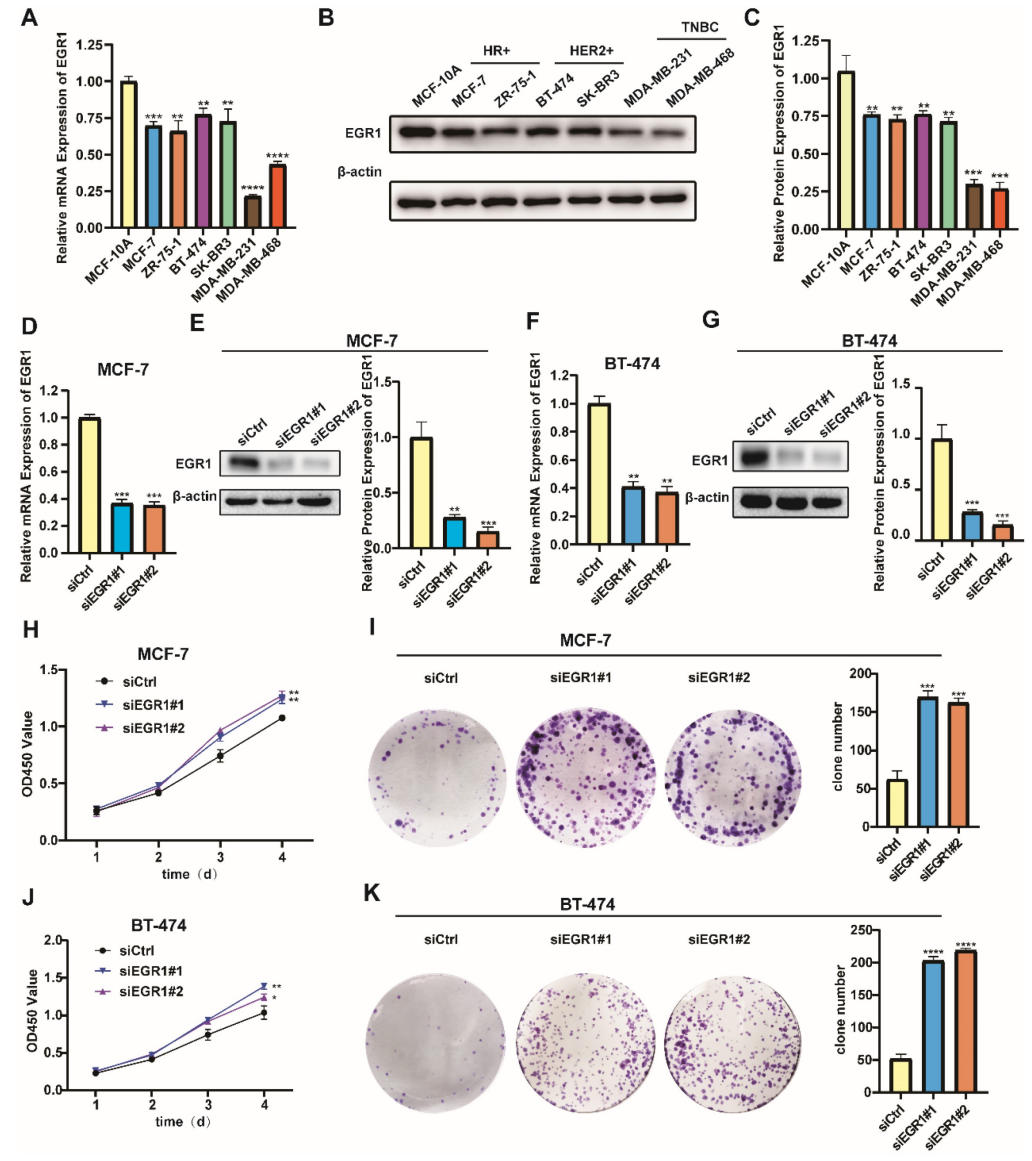
** Knockdown of EGR1 promoted the proliferation of BC cells. (A-C)** The mRNA and protein expression levels of EGR1 in MCF-10A and BC cell lines. **(D, E)** The knockdown efficiency of EGR1 in MCF-7 was detected by qRT-PCR and Western blot. **(F, G)** The knockdown efficiency of EGR1 in BT-474 was detected by qRT-PCR and Western blot. **(H, I)** CCK-8 assay and colony formation experiment in MCF-7 with or without EGR1 downregulation. **(J, K)** CCK-8 assay and colony formation experiment in BT-474 with or without EGR1 downregulation.

Building on our analysis, we further conducted experiments to identify the role of EGR1 in BC cells. Initially, we employed MCF-10A and six different BC cell lines to detect the mRNA and protein expression of EGR1 using qRT-PCR and Western blot assay. Compared with those in MCF-10A, the mRNA and protein expression of EGR1 in breast cancer cell lines were decreased (Figure 2A-C). Then we established EGR1 knockdown MCF-7 and BT-474 cells to understand the effect of EGR1 on BC cells (Figure 2D-G). As shown by CCK-8 assay, the OD450 values of both cells were increased by EGR1 downregulation (Figure 2H and J). This observation was also consistent with the increased numbers of colonies in both cells due to downregulated EGR1 (Figure 2I and K). Collectively, these data suggested EGR1 knockdown promoted the proliferation of BC cells.

### Overexpression of EGR1 suppressed the proliferation of BC cells

**Figure 3:**
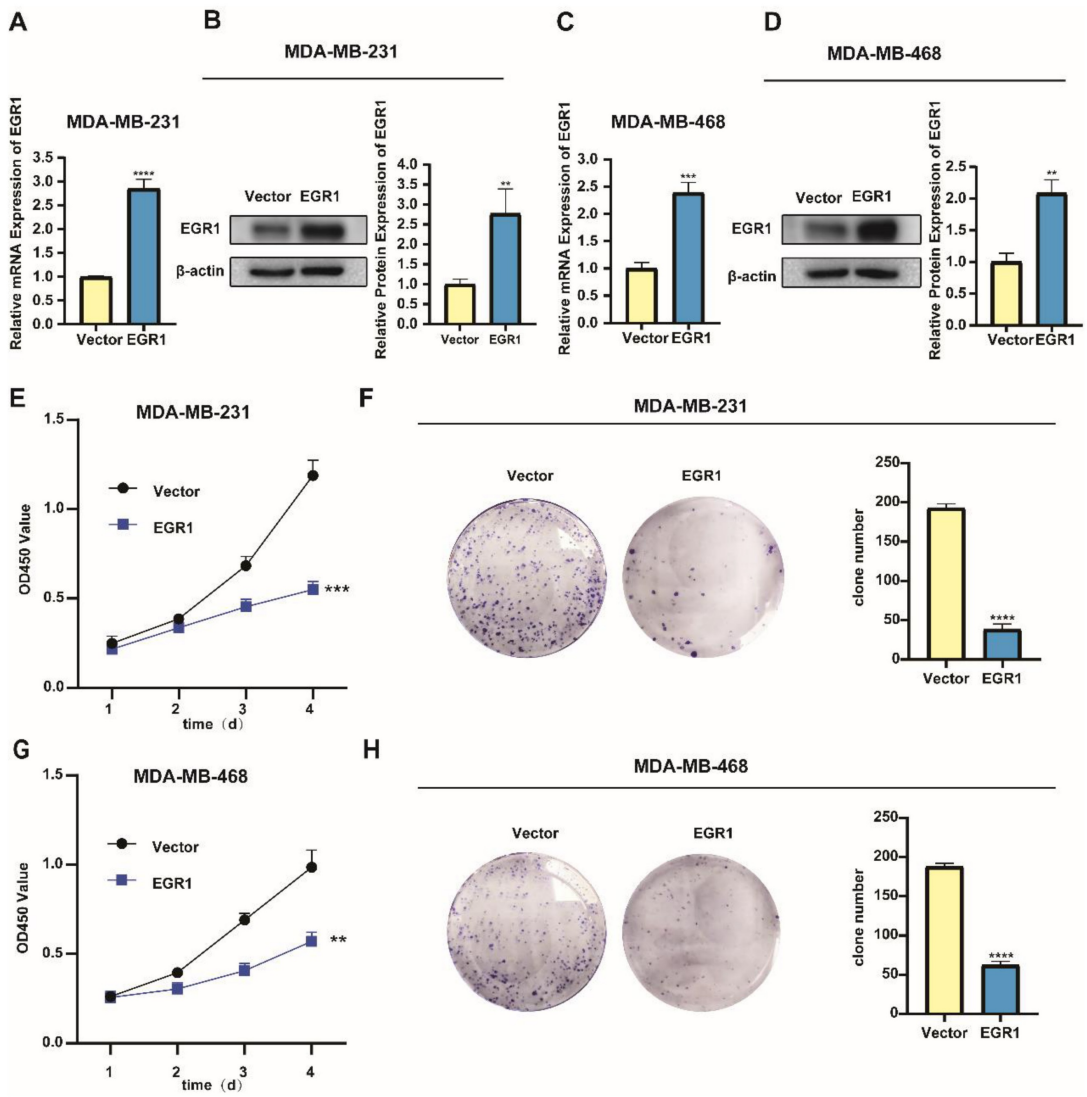
** Overexpression of EGR1 suppressed the proliferation of BC cells. (A, B)** The expression levels of EGR1 mRNA and protein in MDA-MB-231 with or without EGR1 overexpression were detected by qRT-PCR and Western blot. **(C, D)** The expression levels of EGR1 mRNA and protein in MDA-MB-468 with or without EGR1 overexpression were detected by qRT-PCR and Western blot. **(E, F)** CCK-8 assay and colony formation experiment in MDA-MB-231 with or without EGR1 upregulation. **(G, H)** CCK-8 assay and colony formation experiment in MDA-MB-468 with or without EGR1 upregulation.

In addition, we noticed lower levels of mRNA and protein expression of EGR1 in the triple-negative breast cancer (TNBC) cell lines MDA-MB-231 and MDA-MB-468 (Figure 2A-C). Subsequently, we successfully established MDA-MB-231 and MDA-MB-468 cells with overexpression of EGR1 (Figure 3A-D). Through CCK-8 assay and colony formation assay, we found that cells with overexpression of EGR1 exhibited lower OD450 values and less colony formation (Figure 3E-H). In summary, these data suggested EGR1 overexpression inhibited the proliferation of BC cells.

### EGR1 knockdown alleviated erastin-induced ferroptosis in BC cells

**Figure 4:**
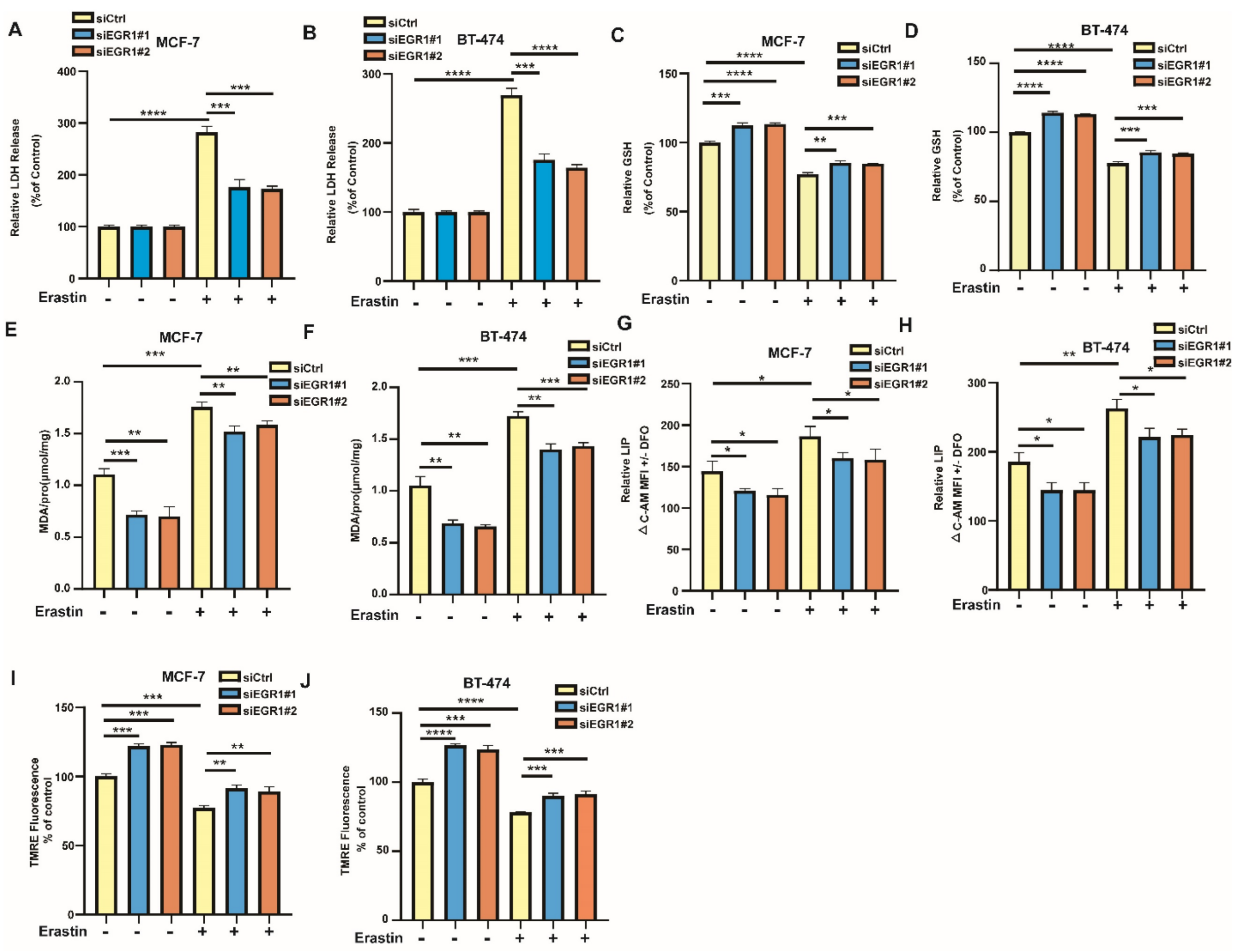
** EGR1 knockdown alleviated erastin-induced ferroptosis in BC cells. (A, B)** LDH release levels in parental and EGR1 knockdown MCF-7 and BT-474 cells treated with or without ferroptosis inducer erastin were detected by LDH release assay. **(C, D)** GSH levels in parental and EGR1 knockdown MCF-7 and BT-474 cells treated with or without ferroptosis inducer erastin were detected by MCB assay. **(E, F)** MDA levels in parental and EGR1 knockdown MCF-7 and BT-474 cells treated with or without ferroptosis inducer erastin were detected by MDA assay kit. **(G, H)** LIP levels in parental and EGR1 knockdown MCF-7 and BT-474 cells treated with or without ferroptosis inducer erastin were detected by C-AM assay. **(I, J)** Mitochondrial membrane potential levels in parental and EGR1 knockdown MCF-7 and BT-474 cells treated with or without ferroptosis inducer erastin were detected by TMRE assay.

Additionally, we examined the changes of ferroptosis-related markers in EGR1 knockdown MCF-7 and BT-474 cells. By LDH release assay, we found that LDH release levels were increased in parental cells by ferroptosis inducer erastin, which was partially alleviated in cells with EGR1 knockdown (Figure 4A and B). Then, we employed MCB assay to measure the alterations in glutathione (GSH) levels between parental and EGR1-deficient cells and evaluated whether downregulation of EGR1 alleviates the decline in intracellular GSH caused by erastin. The results indicated that the downregulation of EGR1 significantly promoted intracellular GSH accumulation and partially reversed the decline in intracellular GSH caused by erastin (Figure 4C and D). Subsequently, we used MDA assay, C-AM assay and TMRE assay to detect the levels of other ferroptosis-related markers, including malondialde-hyde (MDA) levels, labile iron pool (LIP) levels, and mitochondrial membrane potential in parental and EGR1-deficient cells. As expected, knockdown of EGR1 significantly reduced intracellular MDA and LIP accumulation, and enhanced the levels of mitochondrial membrane potential (Figure 4E-J). Moreover, the downregulation of EGR1 partially alleviated the alterations of MDA levels (Figure 4E and F), LIP levels (Figure 4G and H), and mitochondrial membrane potential (Figure 4I and J) caused by erastin. Collectively, these results indicated that downregulation of EGR1 alleviated the occurrence of ferroptosis induced by erastin in BC cells.

### Overexpression of EGR1 facilitated erastin-induced ferroptosis in BC cells

**Figure 5:**
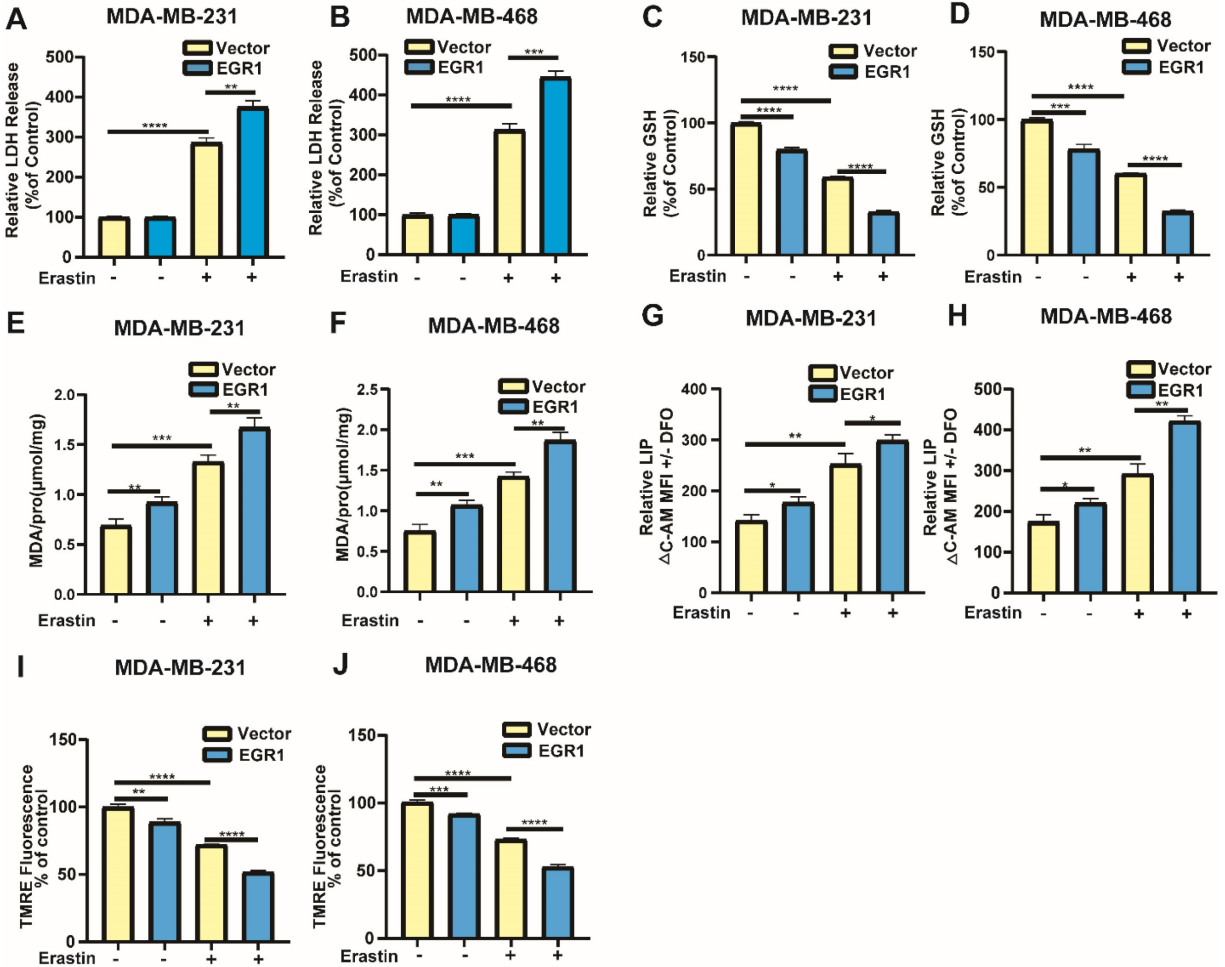
** Overexpression of EGR1 facilitated erastin-induced ferroptosis in BC cells. (A, B)** LDH release levels in parental and EGR1-upregulated MDA-MB-231 and MDA-MB-468 cells treated with or without ferroptosis inducer erastin were detected by LDH release assay. **(C, D)** GSH levels in parental and EGR1-upregulated MDA-MB-231 and MDA-MB-468 cells treated with or without ferroptosis inducer erastin were detected by MCB assay. **(E, F)** MDA levels in parental and EGR1-upregulated MDA-MB-231 and MDA-MB-468 cells treated with or without ferroptosis inducer erastin were detected by MDA assay kit. **(G, H)** LIP levels in parental and EGR1-upregulated MDA-MB-231 and MDA-MB-468 cells treated with or without ferroptosis inducer erastin were detected by C-AM assay. **(I, J)** Mitochondrial membrane potential levels in parental and EGR1-upregulated MDA-MB-231 and MDA-MB-468 cells treated with or without ferroptosis inducer erastin were detected by TMRE assay.

Building on the above results, we further investigated whether markers associated with ferroptosis exhibited changes in cells with overexpression of EGR1. Similarly, through LDH release assay, we found that overexpression of EGR1 increased the LDH release levels in cells treated with erastin compared with parental cells treated with erastin (Figure 5A and B). Then, we employed MCB assay to detect GSH levels in MDA-MB-231 and MDA-MB-468 cells with or without EGR1 overexpression. The results showed that overexpression of EGR1 inhibited intracellular GSH accumulation and enhanced the effect that GSH levels were reduced caused by erastin (Figure 5C and D). Subsequently, we employed MDA assay, C-AM assay and TMRE assay to measure the levels of MDA, LIP and mitochondrial membrane potential. As expected, the intracellular MDA and LIP dramatically accumulated in MDA-MB-231 and MDA-MB-468 cells with overexpression of EGR1, and mitochondrial membrane potential was declined compared with parental cells. Treatment with erastin increased the levels of MDA and LIP, as well as reduced the levels of mitochondrial membrane potential, which was further promoted by EGR1 overexpression (Figure 5E-J). Thus, these data demonstrated that overexpression of EGR1 promoted erastin-induced ferroptosis in BC cells.

### EGR1 regulated the phosphorylation of Nrf2 and promoted the expression of HMOX1

**Figure 6:**
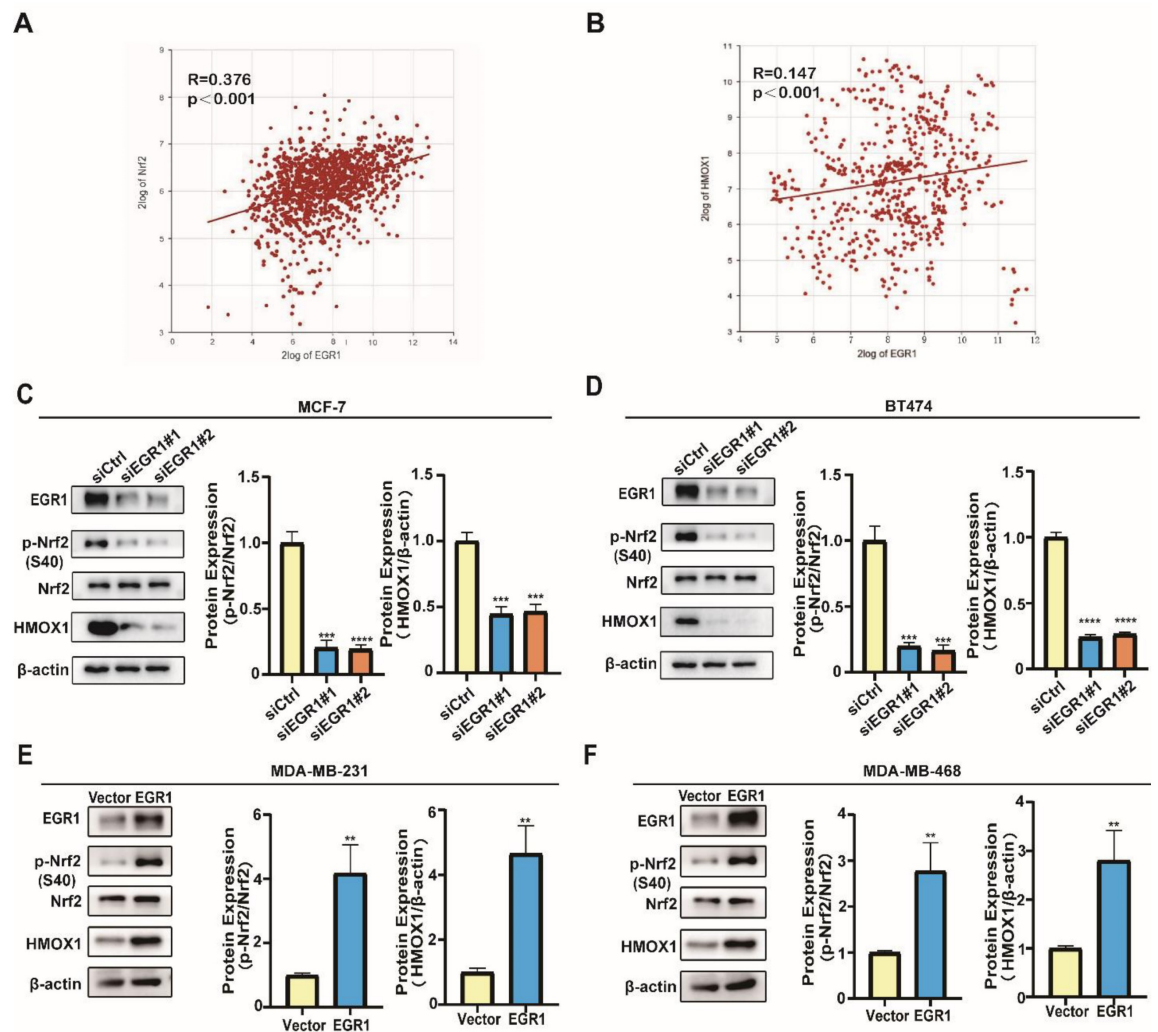
** EGR1 regulated the phosphorylation of Nrf2 and promoted the expression of HMOX1. (A)** Correlation analysis of EGR1 and Nrf2 mRNA expression levels in BC tissues from R2 online database. **(B)** Correlation analysis of EGR1 and HMOX1 mRNA expression levels in BC tissues from R2 online database. **(C, D)** Western blot for ferroptosis related proteins (p-Nrf2, Nrf2, HMOX1) in MCF-7 and BT-474 cells with or without EGR1 knockdown. **(E, F)** Western blot for ferroptosis related proteins (p-Nrf2, Nrf2, HMOX1) in MDA-MB-231 and MDA-MB-468 cells with or without EGR1 overexpression.

In order to elucidate the molecular mechanism, we further explored the signaling pathway through which EGR1 regulated ferroptosis in BC cells. By analyzing data obtained from the R2 online public database, we found a positive correlation between the expression of EGR1 and the key molecules Nrf2 and HMOX1 in the ferroptosis pathway (Figure 6A and B). Subsequently, we employed Western blot assay to evaluate the changes in the phosphorylation levels of Nrf2 and the expression of HMOX1 in cells with upregulation or downregulation of EGR1. We found that the phosphorylation levels of Nrf2 and the expression of HMOX1 decreased in EGR1 knockdown MCF-7 and BT-474 cells compared with those in parental cells (Figure 6C and D). Furthermore, in MDA-MB-231 and MDA-MB-468 cells with overexpression of EGR1, both the phosphorylation levels of Nrf2 and the expression of HMOX1 increased compared with those in parental cells (Figure 6E and F). Collectively, we found that EGR1 regulated the phosphorylation of Nrf2 and promoted the expression of HMOX1 in BC cells.

### EGR1 facilitated erastin-induced ferroptosis by activating Nrf2-HMOX1 signaling pathway

**Figure 7:**
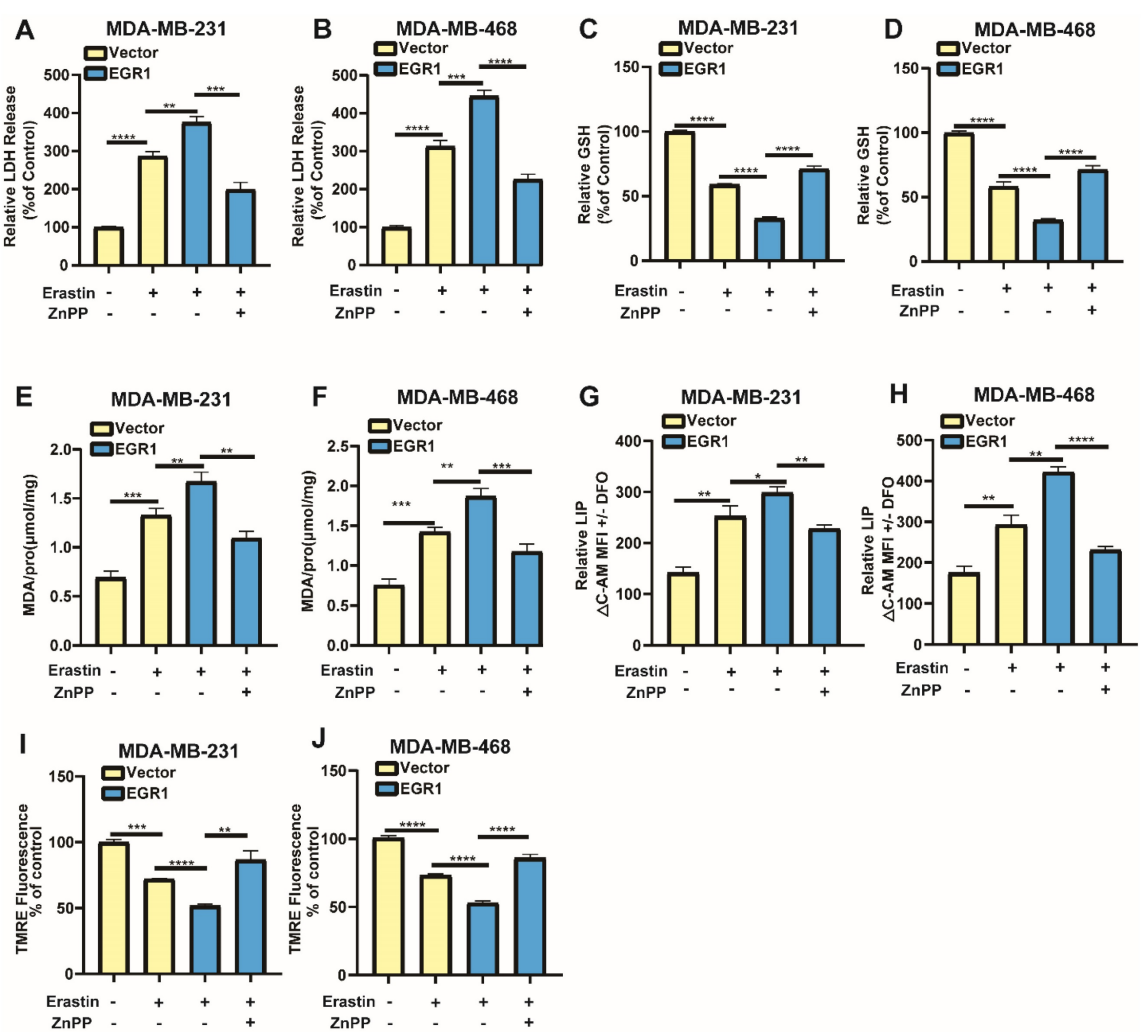
** EGR1 facilitated erastin-induced ferroptosis by activating Nrf2-HMOX1 signaling pathway. (A, B)** LDH release levels in parental and EGR1-upregulated MDA-MB-231 and MDA-MB-468 cells treated with vehicle or erastin and the rescue assay with ZnPP. **(C, D)** GSH levels in parental and EGR1-upregulated MDA-MB-231 and MDA-MB-468 cells treated with vehicle or erastin and the rescue assay with ZnPP. **(E, F)** MDA levels in parental and EGR1-upregulated MDA-MB-231 and MDA-MB-468 cells treated with vehicle or erastin and the rescue assay with ZnPP. **(G, H)** LIP levels in parental and EGR1-upregulated MDA-MB-231 and MDA-MB-468 cells treated with vehicle or erastin and the rescue assay with ZnPP. **(I, J)** Mitochondrial membrane potential levels in parental and EGR1-upregulated MDA-MB-231 and MDA-MB-468 cells treated with vehicle or erastin and the rescue assay with ZnPP.

To confirm whether EGR1 regulated erastin-induced ferroptosis through Nrf2-HMOX1 signaling pathway, we employed Nrf2-HMOX1 signaling pathway inhibitor ZnPP to examined the changes of ferroptosis-related markers in cells with overexpression of EGR1. We found that LDH release levels in EGR1-upregulated cells treated with erastin were increased compared with those in parental cells treated with erastin, which was reversed by the co-treatment of ZnPP (Figure 7A and B). Moreover, the levels of GSH (Figure 7C and D) and mitochondrial membrane potential (Figure 7I and J) in EGR1-upregulated cells treated with erastin were decreased compared with those in parental cells treated with erastin, which was alleviated by the treatment of ZnPP. MDA and LIP levels in EGR1-upregulated cells treated with erastin were increased compared with those in parental cells, which was also reversed by the treatment of ZnPP (Figure 7E-H). Therefore, EGR1 facilitated erastin-induced ferroptosis by activating Nrf2-HMOX1 signaling pathway in BC cells.

### Overexpression of EGR1 facilitated the anti-tumor effect caused by erastin *in vivo*

To confirm the function of EGR1 *in vivo*, we constructed xenograft models by injecting MDA-MB-231 cells with or without EGR1 overexpression into the fat pads of female nude mice treated with or without erastin (i.p., 20 mg/kg, once every other day) and ZnPP (i.p., 10 mg/kg, once every other day). We found that the tumor sizes in mice treated with erastin were reduced compared with those in mice treated with vehicle. Moreover, the tumor sizes in mice bearing EGR1-upregulated cells and treated with erastin were decreased compared with those in mice bearing parental cells and treated with erastin, which was reversed by the treatment of ZnPP (Figure 7A and B). After sacrificing the mice, we measured the final weight of the tumors and obtained the same conclusion (Figure 7C). Furthermore, we analyzed the expression of Ki67 in the tumors using immunohistochemistry staining. The results showed that Ki67 was located in the nucleus. Additionally, Ki67-positive cells were less in mice bearing EGR1-upregulated MDA-MB-231 cells and treated with erastin compared with those in mice bearing parental MDA-MB-231 cells and treated with erastin, which was reversed by the treatment of ZnPP (Figure 7D and E). In summary, these data demonstrated that overexpression of EGR1 enhances the anti-tumor effect caused by erastin *in vivo*.

## Discussion

Ferroptosis has been identified as an important mechanism to inhibit the growth of tumors 19. Erastin has been found to inhibit the growth of BC by inducing ferroptosis 20. However, tumors developed various ways to resist ferroptosis 12. Our study investigated the role of EGR1 in the regulation of proliferation and erastin-induced ferroptosis in BC. Firstly, our study demonstrated the lower expression of EGR1 in BC tissues through analysis of public databases and our clinical samples. Moreover, we revealed the correlation between the expression of EGR1 and the prognosis of BC patients. Similarly, a previous study based on public databases also found that higher expression of EGR1 was associated with longer overall survival and disease-free survival in BC patients 21. However, this study lacked sufficient experimental evidence to elucidate the effect of EGR1 in BC and the underlying mechanism. Therefore, further research is needed to support these conclusions.

To further understand the mechanism of EGR1 in BC, by analyzing the data from R2 database and carrying out GO and KEGG analysis, we revealed that EGR1 was involved in the glutathione metabolism signaling pathway. Previous studies have emphasized the effects of intracellular GSH in proliferation and ferroptosis 22. Therefore, we firstly evaluated its effects on the proliferation of BC cells. We found that overexpression of EGR1 suppressed the proliferation of BC cells. Similarly, previous studies have also demonstrated the inhibitory effect of EGR1 in the growth of papillary thyroid carcinoma, colon cancer and osteosarcoma 23-25. These findings strongly support the inhibitory role of EGR1 in the growth of various tumors, further supporting our data and emphasizing the anti-tumor role of EGR1.

Erastin is known to induce ferroptosis by inhibiting the intracellular accumulation of GSH which serves as a regulator of cellular redox state and protecting cells from death caused by lipid peroxidation 26, 27. Although erastin has been found to suppresses BC by inducing ferroptosis 28, tumors escape ferroptosis through various mechanism 12. Therefore, we evaluated the association between EGR1 and ferroptosis induced by erastin in BC cells. We observed the decline in the levels of GSH and the alterations in other ferroptosis-related markers in cells due to the overexpression of EGR1, which was fundamental for ferroptosis. When the levels of the markers associated with ferroptosis reach a certain range, the cells will undergo ferroptosis due to excess iron 22, 29. Consequently, we found that EGR1 acted as a driver to promote erastin-induced ferroptosis in BC. A previous study has shown that EGR1 overexpression reversed the resistance of bladder cancer cells to ferroptosis inducer, thereby increased their sensitivity to ferroptosis 30. Additionally, another study indicated that the inhibition of EGR1 can reduce ferroptosis in cardiomyocytes by increasing the expression of GPX4^31^. These studies collectively demonstrate that EGR1 enhanced the sensitivity of cells to ferroptosis through different mechanisms. Our study further supports the role of EGR1 in the treatment of BC and provides beneficial evidence for previous studies.

Ferroptosis induction has emerged as a promising therapeutic strategy for cancers 32. Nrf2-HMOX1 signaling pathway, a classic ferroptosis signaling pathway, plays an important role in various diseases 33. For example, Tagitinin C induced ferroptosis in colon cancer cells through the activation of Nrf2-HMOX1 pathway 34. Moreover, ferroptosis induction by Cyclophosphamide through the activation of NRF2-HMOX1 signaling pathway has been found 35. In addition, it has been reported that BAY 11-7085 induced ferroptosis via Nrf2-HMOX1 signaling pathway 36. These studies demonstrate that activating Nrf2-HMOX1 signaling pathway induces ferroptosis in various types of cancer. With these previous findings strongly supporting, our study demonstrated that EGR1 promoted erastin-induced ferroptosis in BC cells by activating Nrf2-HMOX1 signaling pathway.

In conclusion, we confirm the lower expression of EGR1 in BC tissues and the correlation between EGR1 and the prognosis of BC patients. Moreover, through *in vitro* and *in vivo* experiments, we demonstrate that overexpression of EGR1 suppresses the proliferation and facilitates erastin-induced ferroptosis in BC through activating Nrf2-HMOX1 signaling pathway. Furthermore, our research highlights the anti-tumor effect of EGR1 and identifies it as a prognostic indicator and potential therapeutic target for BC treatment.

## Supplementary Material

Supplementary tables.

## Figures and Tables

**Figure 8:**
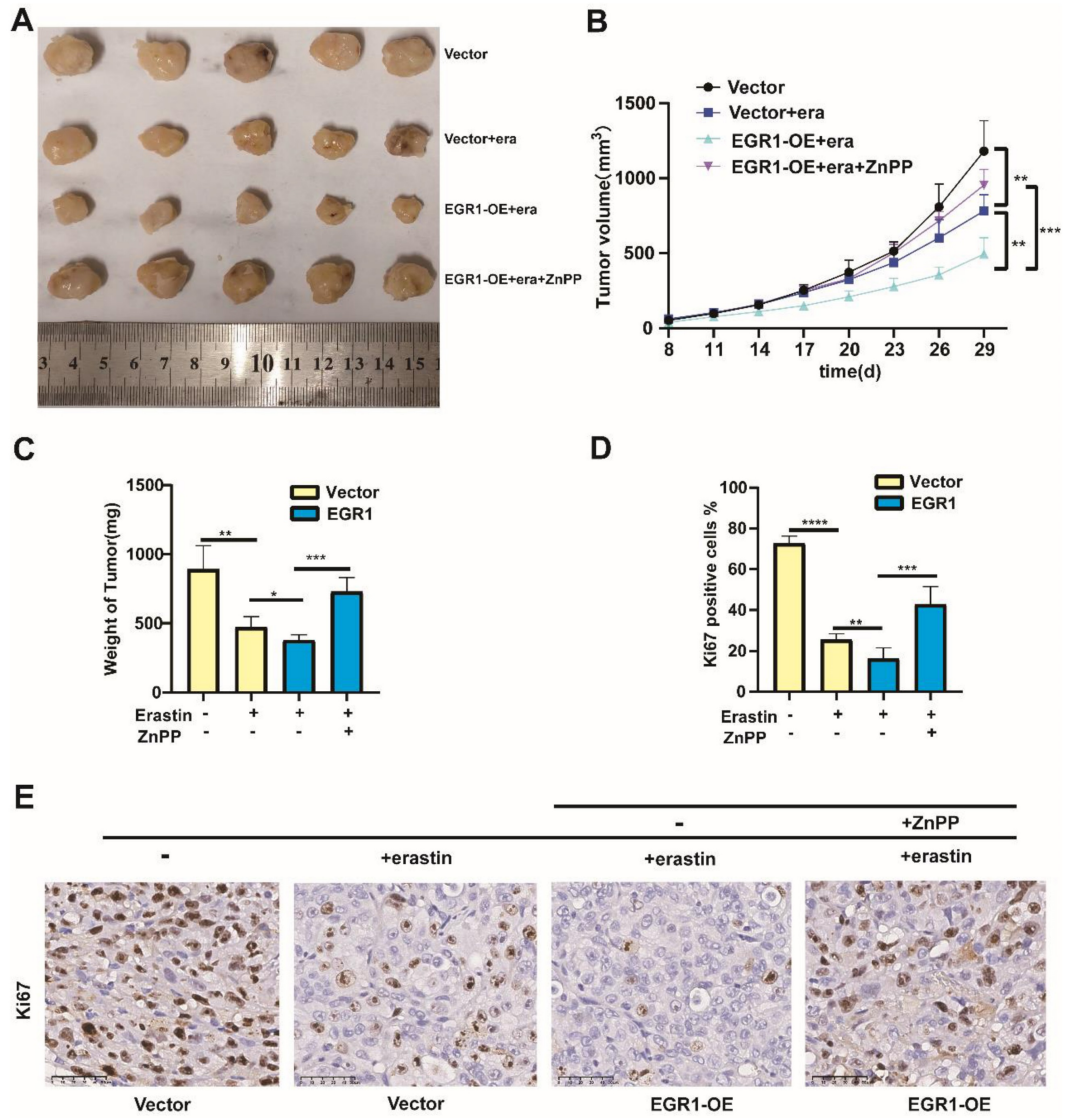
** Overexpression of EGR1 facilitated the anti-tumor effect caused by erastin *in vivo*. (A)** Xenograft models by injecting MDA-MB-231 cells with or without EGR1 overexpression. The nude mice bearing cells were treated with or without erastin (i.p., 20 mg/kg, once every other day) and ZnPP (i.p., 10 mg/kg, once every other day). Representative images of the treated tumors were shown. **(B)** Tumor volume was monitored every 3 days. **(C)** Final weights of tumors were measured on the terminal day. **(D, E)** Statistical analysis of Ki67 expression levels and representative images of Ki67 staining in the tumors.

**Table 1:** clinic parameters of enrolled patient.

Clinic parameters	EGR1 expresssion		*χ^2^*	*p* vaule
	Low	High		
Age			0.43	0.509
<60	7	16		
≥60	6	21		
Grade			2.09	0.351
I	7	13		
II	4	11		
III	2	13		
TNM stage			7.73	0.005
I-II	3	25		
III	10	12		
Subtype			7.27	0.018
TNBC	9	10		
HR+/HER2+	4	27		
Ki-67			4.43	0.034
<20	2	18		
≥20	11	19		
