# Supplementary material for: EGR1 Promotes Erastin-induced Ferroptosis Through Activating Nrf2-HMOX1 Signaling Pathway in Breast Cancer Cells
Source: J Cancer. 2024 Jun 24;15(14):4577–90. doi: 10.7150/jca.95328 (PMC11242347; doi:10.7150/jca.95328)
Supplement: Supplementary file 1 — Supplementary tables. [file jcav15p4577s1.pdf]

EGR1 promotes erastin-induced ferroptosis through activating Nrf2-HMOX1 signaling pathway in breast cancer cells

Zhirong Lin[1#], Zifei liu[1#], Zhilong Pan[1], Yunyi Zhang[1], Xinyu Yang[1], Yaxin Feng[1], Ruihua Zhang[1], Wenfeng Zeng[1], Chang Gong[1], Jianing Chen[1]

1. Breast Tumor Center, Sun Yat-Sen Memorial Hospital, Sun Yat-Sen University, Guangzhou, 510120, China.

Corresponding author：Jianing Chen, E-mail：chenjn335@mail.sysu.edu.cn, Tel: +86-020-34070870; Chang Gong, E-mail: gchang@mail.sysu.edu.cn; Wenfeng Zeng, E-mail: zengwf25@mail.sysu.edu.cn

# Contributed equally.

Table S1 The sequences of primers for the qRT-PCR analysis

Name	Sequence
EGR1-F	5'-CCACGCCGAACACTGACATT-3'
EGR1-R	5'-GAGGGGTTAGCGAAGGCTG-3'
GAPDH-F	5'-CTGGGCTACACTGAGCACC-3'
GAPDH-R	5'-AAGTGGTCGTTGAGGGCAATG-3'

Table S2 The sequences of two sense strands of siRNA targeting EGR1.

Name	Sequence
siEGR1#1	sense: 5'-AGAUGAUGCUGCUGAGCAA-3'
	antisense: 5'-UUGCUCAGCAGCAUCAUCU-3'
siEGR1#2	sense: 5'-GCAGCAGCAGCACCUUCAA-3'
	antisense: 5'-UUGAAGGUGCUGCUGCUGC-3'

Abbreviations:

BC: breast cancer; C-AM: calcein-acetoxymethyl ester; EGR1: early growth response 1; EMT: Epithelial-Mesenchymal Transition; GSH: glutathione; GPX4: glutathione peroxidase 4; HMOX1: heme oxygenase 1; LDH: lactate dehydrogenase; LIP: labile iron pool; MDA: malondialdehyde; Nrf2: NFE2 like bZIP transcription factor; qRT-PCR: quantitative real-time polymerase chain reaction; TCGA: the Cancer Genome Atlas; TMRE: tetramethylrhodamine ethyl ester.
